# Flexible SbSI/Polyurethane Nanocomposite for Sensing and Energy Harvesting

**DOI:** 10.3390/s23010063

**Published:** 2022-12-21

**Authors:** Bartłomiej Nowacki, Jakub Jała, Krystian Mistewicz, Roman Przyłucki, Grzegorz Kopeć, Tomasz Stenzel

**Affiliations:** 1 Department of Materials Technologies, Faculty of Materials Engineering, Silesian University of Technology, Krasińskiego 8, 40-019 Katowice, Poland; 2 Institute of Physics—Center for Science and Education, Silesian University of Technology, Krasińskiego 8, 40-019 Katowice, Poland; 3 Department of Industrial Informatics, Faculty of Materials Science, Silesian University of Technology, Krasińskiego 8, 40-019 Katowice, Poland

**Keywords:** nanocomposite, piezoelectric effect, flexible electronics, energy harvesting

## Abstract

The dynamic development of flexible wearable electronics creates new possibilities for the production and use of new types of sensors. Recently, polymer nanocomposites have gained great popularity in the fabrication of sensors. They possess both the mechanical advantages of polymers and the functional properties of nanomaterials. The main drawback of such systems is the complexity of their manufacturing. This article presents, for the first time, fabrication of an antimony sulfoiodide (SbSI) and polyurethane (PU) nanocomposite and its application as a piezoelectric nanogenerator for strain detection. The SbSI/PU nanocomposite was prepared using simple, fast, and efficient technology. It allowed the obtainment of a high amount of material without the need to apply complex chemical methods or material processing. The SbSI/PU nanocomposite exhibited high flexibility and durability. The microstructure and chemical composition of the prepared material were investigated using scanning electron microscopy (SEM) and energy-dispersive X-ray spectroscopy (EDS), respectively. These studies revealed a lack of defects in the material structure and relatively low agglomeration of nanowires. The piezoelectric response of SbSI/PU nanocomposite was measured by pressing the sample with a pneumatic actuator at different excitation frequencies. It is proposed that the developed nanocomposite can be introduced into the shoe sole in order to harvest energy from human body movement.

## 1. Introduction

Flexible electronic devices possess numerous advantages over the conventional rigid elements. Due to inherent bendability, stretchability, and twistability, flexible electronic devices are attractive for use in medicine [1,2,3,4], energy storage or harvesting [5,6,7,8,9,10], robotics, and smart clothing [11,12,13,14,15,16]. The materials for flexible electronics should exhibit remarkable elasticity along with good resistance to fatigue damage originating from frequent strain or stress influence. Their functional properties may refer to optical, electrical, or magnetic characteristics depending on the relevant application of a material. In case of polymer composites, the functional fillers are dispersed in a flexible matrix to achieve a material with desirable mechanical properties and diminished (in comparison with pristine filler) functional characteristics. A detection of mechanical deformation can be accomplished using three fundamental phenomena: piezoresistive effect, strain-dependent electric capacitance variation, and piezoelectricity [12]. Among them, the piezoelectric effect is the most promising for energy harvesting.

The human body generates a lot of energy through thermal radiation and movement. Until now, various devices have been proposed for biomechanical energy harvesting [17,18,19]. The recent studies in this field are focused on energy recovery from human body motion, friction, vibrations, and radiation absorption [20,21,22,23,24,25,26,27,28,29]. There are several types of approaches to construct energy harvesters. One strategy is to build the complicated device from widely available materials [20,23]. Another approach is to use functional materials, e.g., piezoelectric or photovoltaic compounds. This allows to simplify the method of device manufacturing, whereas the production costs are increased. The significant advantage of such devices is their compact size, which is crucial for application in wearable electronics [21,22,24,25,26,27,28,29]. The hybrid energy harvesters has become more popular in recent years [26,27]. They recover different forms of energy simultaneously in order to achieve a high efficiency of energy harvesting.

Piezoelectric nanogenerators (PENGs) convert mechanical energy into the electrical output. The first piezoelectric nanogenerators were reported in 2006 [16]. Many different materials have been used in flexible PENGs so far, including wurtzite structure materials, perovskite structure materials, 2D piezoelectric materials, organic–inorganic perovskites, polyvinylidene fluoride (PVDF)-based materials, and bio-based piezoelectric materials [5]. Among the mentioned materials, special attention from the scientific community has been paid to ZnO (wurtzite) composites [30,31,32,33,34], lead zirconate titanate (PZT), BaTiO_3_, perovskites [35,36,37,38,39], and electrospun PVDF composites [40,41,42].

One of the main disadvantages of most commonly used piezoelectric ceramics is their brittleness. This drawback is avoided in the flexible piezoelectric composites. Various polymers can be used as a flexible matrix of the composite, e.g., polyurethane (PU), polyethylene terephthalate (PET), polydimethylsiloxane (PDMS), and PVDF. Polyurethane has been proved to be a flexible and fatigue-resistant polymer by numerous industry applications, e.g., fabrication of shoe soles, encapsulation of electrical devices, and production of automotive elements. Furthermore, polyurethane has been considered as a promising material for flexible electronics. It has been utilized in capacitive harvesters [43], fabric-based stretchable triboelectric generators [44], piezoelectric harvesters [45], carbon-based conductive flexible composites [46,47,48], strain sensors [49], doped polyurethane foams with excellent piezoelectric properties [50], and piezoelectric harvesters introduced into the road for scavenging energy from passing vehicles [51].

This paper presents, for the first time, an incorporation of piezoelectric nanowires of antimony sulfoiodide (SbSI) into the flexible polyurethane matrix. SbSI possesses a large *d*_33_ piezoelectric coefficient of 10^−9^ C/N [52]. Most commercially used PZT (lead zirconate titanate) have d33 values reported from 0.2 × 10^−9^ C/N to almost 0.7 × 10^−9^ C/N, depending on manufacturing methods and treatment processes [53,54]. Other commonly used piezoelectric material is perovskite BaTiO_3_, in which the piezoelectric coefficient d33 has values from 0.27 × 10^−9^ C/N to as much as 0.788 × 10^−9^ C/N when doped and properly processed [55,56]. SbSI nanowires have been successfully used as fillers/active elements in several different composites [52,57,58,59]. However, an application of an SbSI composite as a flexible sensor has not been presented before. Incorporation of antimony sulfoiodide nanowires into the flexible polyurethane matrix may open new possibilities of use of this material in advanced wearable electronic devices.

## 2. Materials and Methods

The commercially available polyurethane resin HPE 85A (Synthene, Pont-Sainte-Maxence, France) was used to fabricate the SbSI/polyurethane nanocomposite. SbSI nanowires were synthesized using the sonochemical approach described in [60]. Samples were prepared by mechanical mixing of SbSI nanowires with PU resin in 1 to 4 weight ratio (20% SbSI in polymer matrix). The prepared mixture was degassed using a vacuum pump and cast into 3D- printed molds. The width and length of each of the 5 samples were equal to 35 mm. The thickness of the samples was 1 mm and 2 mm. Gold electrodes with a thickness of 150 nm were deposited onto samples using a Q150ES rotary-pumped coater (Quorum Technologies Ltd., Lewes, UK). The copper wires were attached to the electrodes of the samples using conductive silver paste.

**Figure 1:**
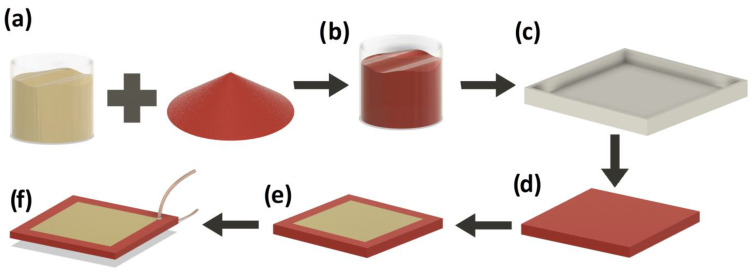
The stages of sample preparation: (**a**) mixing of PU resin and SbSI nanowires, (**b**) mixture degassing, (**c**) casting into the mold, (**d**) extraction of the cured sample from the mold, (**e**) deposition of gold electrodes, and (**f**) attachment of the copper wires.

**Figure 2:**
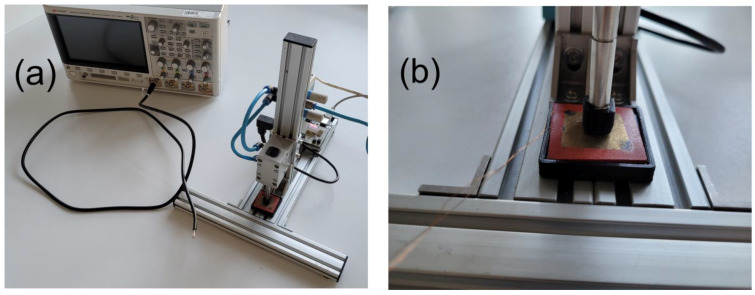
The photographs of (**a**) entire measurement setup and (**b**) sample clamped in the holder.

The sample fabrication was presented in Figure 1. Two samples were subjected to electric poling by applying a voltage of 1.1 kV for 60 min. The dynamic mechanical load was provided by a pneumatic system shown in Figure 2. The operation of the system is presented in the video added as Supplementary Material.

Electrical measurements were conducted using DSOX3104T oscilloscope (Keysight Technologies, Santa Rose, CA, USA). The examination of samples was carried out with three loading frequencies of 1 Hz, 3 Hz, and 7 Hz. The morphology of the SbSI nanowires was studied at the acceleration voltage of 15 kV using a Phenom Pro X (Thermo Fisher Scientific, Waltham, MA, USA) scanning electron microscope (SEM). The measurement data were analyzed using Origin software (OriginLab, Northampton, MA, USA).

## 3. Results and Discussion

### 3.1. Microstructure

**Figure 3:**
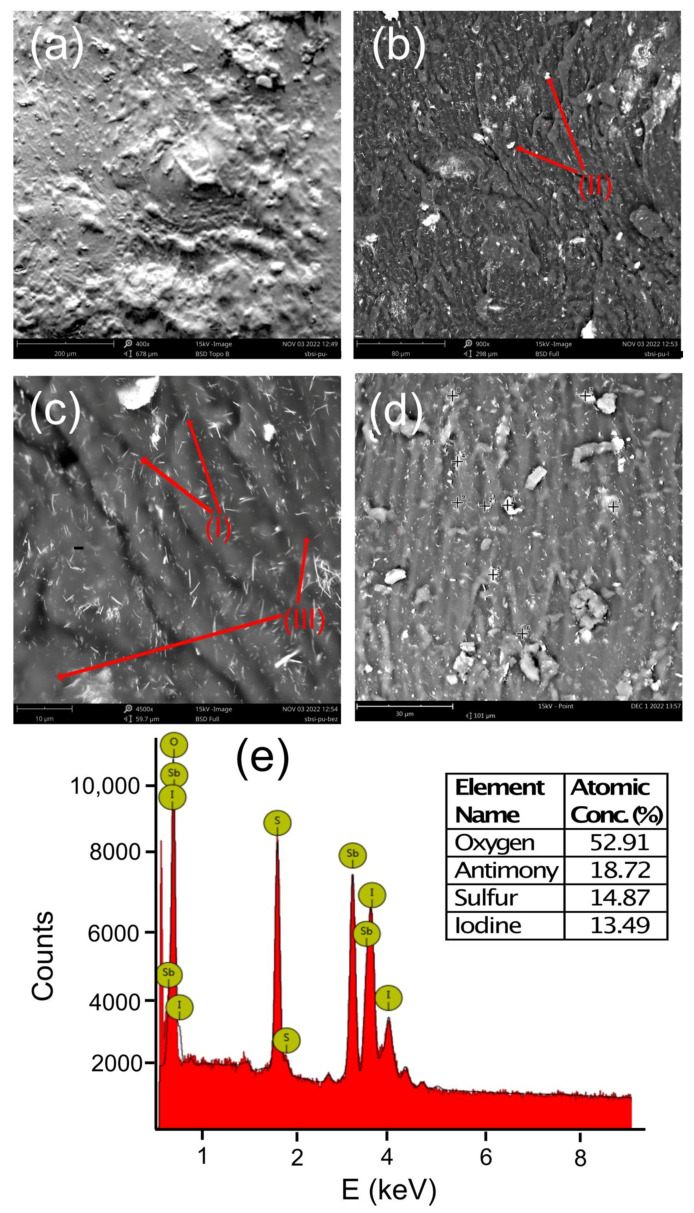
SEM images of (**a**) topology and (**b**–**d**) morphology of the SbSI/PU nanocomposite and (**e**) its EDS spectrum. The labels (I), (II), and (III) in Figures (**b**,**c**) indicate separate SbSI nanowires, agglomerations of SbSI nanowires, and the polyurethane matrix, respectively. The inset in Figure (**e**) presents the table with atomic concentrations of chemical elements detected in the sample.

The sample’s microstructure was examined across the electrodes. The SEM images were acquired using full backscattered electron detector (BSD) and topology BSD modes. This allowed to determine the distribution of nanowires, their agglomerations, and topologies inside the SbSI/PU nanocomposite. Typical SEM images of the sample are presented in Figure 3. The topology (Figure 3a) of the SbSI/PU nanocomposite shows the structure resulting from joining the polymer with SbSI nanowires and cutting the sample. No air bubbles were detected inside the prepared samples. An occurrence of such defects is a common problem in the fabrication of nanocomposites [61,62,63,64].

The absence of air bubbles is extremely advantageous taking into account the short mixing, deagglomeration, and deaeration time of the resin. The short time of deagglomeration could also have a positive effect on this aspect. It is extremely important due to elasticity and hardness of the final product. The lack of air bubbles also positively affects the quality of the nanocomposite itself. Air bubbles would also have an adverse effect on the piezoelectric response. Air bubbles are electrical insulators. It could attenuate the piezoelectric signal. This allows for maintaining the hardness value of 85 in Shore A scale, which is guaranteed by the resin manufacturer. The lack of air bubbles may be due to the used polymer and the relatively favorable flow properties of the resin. This aspect is extremely beneficial for a mass production.

The short manufacturing time will allow to produce more nanocomposite with lower energy consumption. Figure 3b,c present the structure morphologies. Figure 3 shows the nanocomposite with SbSI nanowires with diameters of about 10–50 nm and lengths of several micrometers [60]. The agglomerates’ appearance can be observed. The agglomerates are relatively evenly distributed in the sample. They are not of a large size, which is a common problem in the preparation of nanocomposites. In the case of the 20% weight nanofiller concentration, the nanowires were dispersed uniformly in the polymer matrix. This is an extremely important feature taking into consideration the insulating properties of the matrix. Too large spaces without nanowires could lead to high electrical resistance of the nanocomposite.

The random orientation of the SbSI nanowires was observed (Figure 3c) suggesting isotropic properties of the nanocomposite. It was found that the arrangement of the nanowires did not depend on the direction of pouring the resin. It allowed to obtain the homogeneity of the entire nanocomposite and repeatability of the sample fabrication.

The SbSI/PU nanocomposite was also investigated using energy dispersive spectroscopy (EDS). The examined area of the sample and corresponding EDS spectrum are shown in Figure 3d,e, respectively. A slight excess of antimony was observed, which is the residue from the synthesis that was not removed during rinsing of the material.

### 3.2. Piezoelectric Response

The piezoelectric response of a sample with a thickness of 1 mm and 2 mm to excitation by a pneumatic actuator at frequencies of 1 Hz, 3 Hz, and 7 Hz was investigated. The pneumatic actuator was excited with a pressure of 6 atm. It generated a force of 0.37 N. The work of the system is presented in Supplementary Materials. The differences in the sample response before and after electric poling were investigated. Electrical poling is a popular method of improving the piezoelectric properties of nanocomposites [65,66]. In this work, the influence of the electrical poling parameter in a nanocomposite using SbSI was examined for the first time.

**Figure 4:**
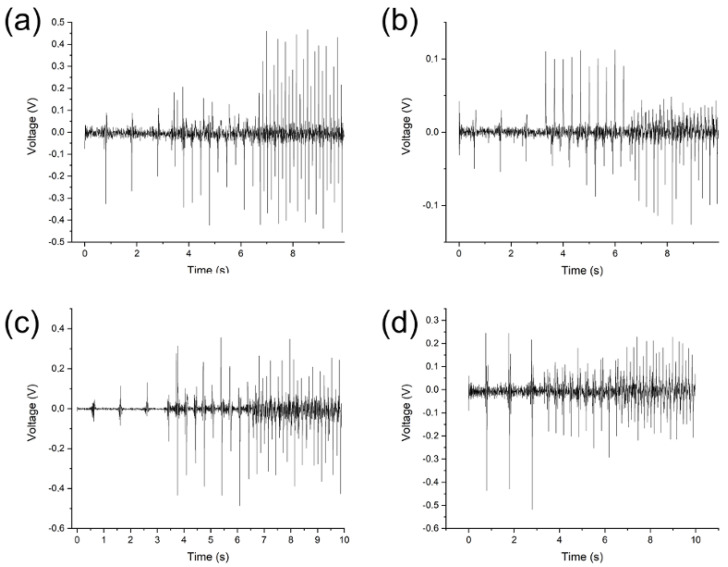
The piezoelectric response registered at various excitation frequencies for different samples of the SbSI/PU nanocomposite: 1 mm thick non-poled (**a**) and poled (**b**) samples; 2 mm thick non-poled (**c**) and poled (**d**) samples.

**Figure 5:**
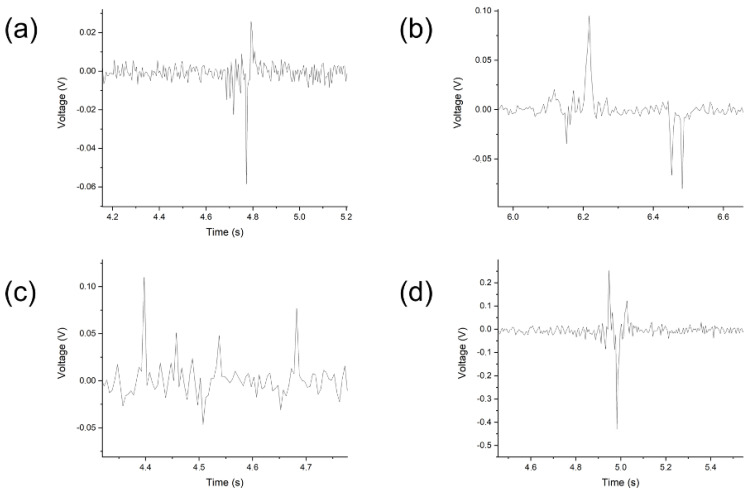
The piezoelectric response registered for different frequencies of the SbSI/PU nanocomposite: poling (**a**–**c**) and non-poling (**d**); 1 Hz (**a**,**d**); 3 Hz (**b**); 7 Hz (**c**).

The typical voltage responses of the SbSI/PU nanocomposite are presented in Figure 4 and Figure 5. The voltage waveforms were filtered in the frequency range from 45 Hz to 55 Hz in order to remove the noise coming from the power supplies of the laboratory equipment. Figure 4a–c show that the voltage increases with increasing frequency. This is a characteristic behavior of the systems based on the piezoelectric effect. In Figure 4d, the highest magnitude of the voltage was measured for the lowest excitation frequency. This may be due to the effect of high electric voltage forming on the polymer.

The significant differences in the characteristics of the piezoelectric response can be noticed comparing Figure 5a,d. Electric poling led to an increase in the reproducibility of the sample response. Simultaneously, it reduced the amplitude of the voltage signal. The conducted research did not allow to clearly define the physical/chemical mechanism of these changes. It is suggested that this may be due to two mechanisms. The first one is the significantly accelerated aging of polyurethane. The second mechanism can be related with the degradation of the polymer around the SbSI nanowires due to significant discharges at the polymer–nanowire interface [67,68,69,70]. It should be underlined that increased reproducibility of the voltage response is beneficial for the application of the material as a reliable sensor. The examined samples showed no fatigue after repeated excitation.

Figure 5 depicts the voltage responses of the SbSI/PU nanocomposite to the single excitation. The vibrations of the material occurred due to its high flexibility. This resulted in a series of peaks, rather than the typically encountered single voltage peak. The disappearance of the voltage peaks originated from the damping properties of the polymer.

The samples vibrated continuously at a higher frequency leading to generation of the voltage pulses. This behavior of the sample is beneficial when it is used as an energy harvester. Figure 5a,b show significant changes in the course of the system response when the system is changed.

The 2 mm thick system is damping. The system gradually relaxes the vibrations. The first peak is smaller than the second, which is probably due to the occurrence of partial compression of the polyurethane during the impact of the actuator.

It is possible to compare the properties of the matrix based on tests with the same piezoelectric material. The polymer matrix shows better mechanical properties in comparison with the cellulose matrix. However, due to the inferior electrical properties of polymer, the voltage signal of the SbSI/PU sensor is 10–100 times smaller than that reported for a sensor based on SbSI/cellulose composite [52]. The magnitude of the piezoelectric response is similar to other piezocomposites with a polymer matrix [71].

Compared with other piezoelectric nanocomposites, the developed sensor performs similarly or slightly worse [71,72,73,74]. This is partly due to the much lower operating frequencies of the sensor. This choice results from the desire to use the material in the sole of shoes in the future. The voltage response of the sensor is about 10–100 times worse than the MgO/PVDF sensor [72] and the sensor’s voltage response is about five times worse than Fe-ZnO/PVDF. The Fe-ZnO sensor was tested at much higher frequencies. An additional disadvantage is the expensive PVDF polymer used [73]. The sensor has a similar response to the sensor based on the BaTiO_3_/polymer [74].

## 4. Conclusions

The fabrication of a sensor/energy harvester based on a flexible nanocomposite of SbSI nanowires and polyurethane was presented herein for the first time. The developed method of the nanocomposite preparation is simple, efficient, and suitable for fabrication of a large amount of this material. The SbSI/PU nanocomposite exhibited both high flexibility and remarkable resistance to fatigue damage. These features are promising for a future use of the SbSI/PU nanocomposite in wearable electronics. It is proposed that the developed material can be inserted into the shoe sole in order to act as an energy harvester and vibration dampener.

The SEM investigations of the SbSI/PU nanocomposite microstructure revealed that the material did not contain air bubbles, which allows to maintain the hardness parameter at a level similar to the polymer resin manufacturer’s data. This will allow future development of vibration-damping energy harvesters or self-measuring damping circuits. A presence of small agglomerates of the SbSI nanowires in the nanocomposite was observed. This may be due to the short deagglomeration time. However, it did not affect the piezoelectric properties of the material since the SbSI nanowires were almost uniformly dispersed in the polymer matrix. Furthermore, the SbSI nanowires were randomly oriented which ensured the isotropy of the prepared nanocomposite.

Piezoelectric responses of samples to mechanical excitation were investigated in various configurations. The influence of sample thickness, the electric poling, and excitation frequency on the piezoelectric response was examined. The thickness of the sample significantly affected the signal value. In some cases, doubling the thickness led to an increase in the voltage response by an order of magnitude. Electric poling reduced the value of the voltage response and increased its repeatability. This is extremely important for sensing purposes where molding can be beneficial. Generally, an increase in the excitation frequency led to an enhancement of the voltage amplitude. The mechanical vibrations in polyurethane were significantly damped. Depending on the additional electric poling step, the SbSI/PU nanocomposite can be successfully applied as a sensor or mechanical energy harvester.

## Data Availability

Available on request.

## Supplementary Materials

The following supporting information can be downloaded at: https://www.mdpi.com/article/10.3390/s23010063/s1.
